# Reply to Kukucka: Calculating error rates in forensic handwriting examiner decisions

**DOI:** 10.1073/pnas.2217508119

**Published:** 2022-12-19

**Authors:** R. Austin Hicklin, Linda Eisenhart, Nicole Richetelli, Peter Belcastro, Ted M. Burkes, Michael Smith, JoAnn Buscaglia, Rebecca Schwartz Perlman, Eugene M. Peters

**Affiliations:** ^a^Noblis, Inc., Reston, VA 20191; ^b^Federal Bureau of Investigation Laboratory, Quantico, VA 22135; ^c^Ideal Innovations, Inc., Arlington, VA 22203

We appreciate the opportunity to respond to the review of our work (1) provided in Kukucka (2) and offer the following responses and clarifications:

*“First, despite characterizing inconclusive decisions as ‘neither correct nor incorrect,’ the authors effectively counted them as correct…”*

Examiners’ performance is multi-dimensional. Inconclusive decisions do not count as errors in calculating false positive/negative rates, but they also do not count as correct in calculating true positive/negative rates. Kukucka bases his assertion on a paper (3) that has been refuted (4, 5). The rate Kukucka suggests (“FPR_CALLS_”) is one of many rates we report: Appendix F1 discusses the varied means of handling inconclusive decisions in conclusion rates.

*“Second… include both ‘definite’ and ‘probable’ judgments in the error rate numerator”*

In our study and in operational casework, forensic examiners explicitly differentiate between levels in conclusion scales to convey different strengths of conclusions or weights of evidence. There is no single error rate: we present four distinct rates of incorrect decisions by which examiners can be assessed in terms of accuracy, as well as four distinct rates of correct decisions by which examiners can be assessed in terms of effectiveness. Blurring such explicit distinctions oversimplifies the results and may be misleading.

*“…raises the false positive rate to 8.2%”*

We report the rate Kukucka suggests (“FPR + IAR_CALLS_”) in Appendix F2, but the correct value is 9.3%.

*“…definitive and qualified conclusions have an equivalent impact on juror decision-making…”*

Kukucka’s references (6, 7) do not support this assertion.

*“Third, many examiners simply declined to compare some of the assigned sets…”*

This is incorrect: participants were unable to choose which comparisons to complete. We controlled the order of assignments so that difficulty and sample attributes would be representative of the whole even if only half of the assigned samples were completed. If participants could have chosen “easy” comparisons and omitted “difficult” ones, we agree that this point might be relevant, but that is not how the test was actually designed or conducted.

*“…under relatively ‘ideal’ conditions…”*

There is no basis for this assertion. A supermajority of participants assessed the samples as a representative of casework. Many factors increased the relative level of difficulty compared to casework: no originals were provided, close nonmates were methodically selected and included twins, and some samples were of limited length and comparability. Kukucka’s assertion cites a paper (8) containing numerous errors and misrepresentations, detailed in refs. (9–11).

*“I fear that its purported error rate could dangerously mislead stakeholders…”*

Reducing complex results to a single number can indeed be misleading. Kukuka essentially selects a method resulting in a high error rate by reducing the denominator and combining distinct categories in the numerator. This is one of several ways of summarizing the results; we are concerned that Kukucka represents this as a sole error rate. We present the results of our research in an empirical, transparent manner. By providing data with a variety of metrics, readers have flexibility in interpreting and applying results in a manner specific to their needs.

## Disclaimer

This is publication number 23.05 of the FBI Laboratory Division. Names of commercial manufacturers are provided for identification purposes only and inclusion does not imply endorsement of the manufacturer or its products or services by the FBI. This work was funded by the FBI Laboratory Division; Ideal Innovations and Noblis were funded under a contract award to Ideal Innovations Inc. from the FBI Laboratory. The views expressed are those of the authors and do not necessarily reflect the official policy or position of the FBI or the US Government.
